# The structure and function of centriolar rootlets

**DOI:** 10.1242/jcs.258544

**Published:** 2021-08-16

**Authors:** Robert Mahen

**Affiliations:** The Medical Research Council Cancer Unit, University of Cambridge, Hills Road, Cambridge CB2 0XZ, UK

**Keywords:** Centrosome, Cilia, Cytoskeleton, Mechanobiology, Organelle assembly, Rootlets

## Abstract

To gain a holistic understanding of cellular function, we must understand not just the role of individual organelles, but also how multiple macromolecular assemblies function collectively. Centrioles produce fundamental cellular processes through their ability to organise cytoskeletal fibres. In addition to nucleating microtubules, centrioles form lesser-known polymers, termed rootlets. Rootlets were identified over a 100 years ago and have been documented morphologically since by electron microscopy in different eukaryotic organisms. Rootlet-knockout animals have been created in various systems, providing insight into their physiological functions. However, the precise structure and function of rootlets is still enigmatic. Here, I consider common themes of rootlet function and assembly across diverse cellular systems. I suggest that the capability of rootlets to form physical links from centrioles to other cellular structures is a general principle unifying their functions in diverse cells and serves as an example of how cellular function arises from collective organellar activity.

## Abstract (precis)

**Summary:** This Review discusses the structure and function of enigmatic cytoskeletal fibres termed centriolar rootlets, suggesting that they form physical links between subcellular structures to allow collective organelle function.

## Introduction

Living matter shows remarkable spatiotemporal behaviour in the intracellular environment. Reactions are compartmentalised into spatial locations, termed organelles. However, organelles do not function in isolation, but collectively in groups, to establish the emergent structure and function of cells. Modern imaging, proteomics, structural biology and genetics have uncovered the functions of many isolated cellular structures, and yet understanding how they collectively produce cellular-level properties is still a major challenge.

Cytoskeletal fibres are ubiquitous within the cell, forming complex connections between multiple organelles (Valm et al., 2017). How organelles maintain their dynamic association in different cellular compartments is still poorly understood. Centrioles are microtubule nucleation centres involved in cellular functions including cell division and the formation of hair-like appendages termed cilia. Centrioles orient cellular geometry and polarity through their ability to seed and interact with other structures, such as cilia, the mitotic spindle or the immunological synapse (Bornens, 2012; Douanne et al., 2021; Tang and Marshall, 2012). These functions require precise organelle subcellular positioning – for example, the formation of exactly two spindle poles during mammalian cell division. They also entail physical contact with other cellular structures and organelles, such as the cell membrane during ciliogenesis (Sorokin, 1968).

**Fig. 1.**
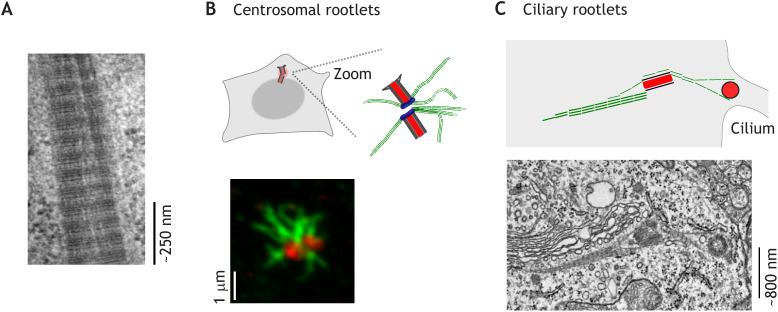
**Centriolar rootlets.** (A) Transmission electron micrograph showing detail of an *Amphioxus* rootlet. Reproduced with permission from Mansfield and Holland (2015), ©2013 The Royal Swedish Academy of Sciences. (B) Immunofluorescent light microscopy and cartoon of rootlets in a non-ciliated human cell. The fluorescence micrograph shows an airyscan confocal image of a human U2OS cell stained with anti-rootletin antibody marking rootlets (green) and anti-NEDD1 antibody marking the pericentriolar material (red). Image by R.M. The cartoon shows a simplified representation of rootlets and two centrioles. (C) Electron micrograph and cartoon of rootlets at a primary cilium in a neuron of the mouse visual cortex. Note that the cilium is not visible on the electron micrograph. Electron microscopy image provided by Dr Carolyn Ott (HHMI Janelia Research Campus), from a publicly available dataset Bock et al. (2011). Rootlets are shown in green and centrioles are shown in red in the schematics.

As well as nucleating microtubules, centrioles form other, lesser-known types of cytoskeletal protein fibres, called rootlets. Rootlets were first described by Engelmann in the 19th century (Engelmann, 1880; Fawcett and Porter, 1954). Decades of electron microscopy since has detailed rootlets as longitudinally aligned filaments with cross-banded striations – less iconic than the centriolar barrel, but arguably as striking (Fig. 1A) (Fawcett and Porter, 1954). Theories on the functions of rootlets include them being absorbers of mechanical stress (Fawcett and Porter, 1954; Gibbons, 1961) or acting as pathways for subcellular traffic (Fariss et al., 1997). In specialized human cell types, such as photoreceptors, rootlets can be among the largest cellular structures (Gilliam et al., 2012; Spira and Milman, 1979). Despite these considerations, mechanistic understanding of the structure and function of rootlets is still enigmatic, particularly in human cells.

Here, I consider common functional themes shared by rootlets in diverse cellular systems. I examine three main groups of rootlet function, relating to: (1) forming physical links as part of multiciliary arrays, (2) mechanosensation in specialised cilia, and (3) maintaining centrosome cohesion (see Glossary) in non-ciliated cells. I suggest that the capability of rootlets to connect centrioles to other cellular structures is a general model for their function and discuss emerging mechanisms by which this might occur in human cells. Throughout, I examine the implications of these considerations for how the intracellular environment self-organises to allow collective organelle function. To these ends, I do not provide comprehensive accounts of every rootlet type, either morphologically or molecularly, but instead direct readers to the primary literature where appropriate.

Glossary **Ciliopathy**: disorders relating to ciliary disfunction.

**Centrosome cohesion**: the close spatial proximity maintained by mature centrioles in many cell types.

**Centrosome disjunction**: splitting of mature centrioles prior to mitosis.

**Chordotonal organ**: invertebrate sensory structure consisting of both mechanosensory and supporting cells, used for a variety of sensory functions including proprioception.

**Cilium power stroke:** movement of a cilium against the surrounding medium with force.

**Intraflagellar transport:** bidirectional movement along ciliary axonemal microtubules.

**Metachronal beating**: sequential wave-like motion of multiple cilia.

**Planar polarity:** orientation of structures within a two-dimensional plane of a tissue.

**Proprioception:** the sense of body movement or position.

**Reptation**: theory from polymer physics describing the movement of very long entangled macromolecules.

## Diversity of rootlet structure and composition

Centrioles are barrel-shaped microtubule-based structures that form the core of centrosomes. Centrioles have multiple different appendages (see Box 1 for an account of mammalian centrosome components and associated terminology). Rootlets are fibrous and often cross-striated cytoskeletal structures extending from centrioles.

**Box 1. Centriolar appendages and associated structures**

**Basal body**: modified centrioles forming the base of a cilium, which influence the orientation of ciliary beating.

**Basal feet**: conical structure at the basal body, also known as subdistal appendages depending on the biological context.

**Centriole:** barrel-shaped microtubule-based structures at the centre of centrosomes and basal bodies.

**Centrosome:** microtubule-organising centre formed from centrioles.

**Cilium**: hair-like structure used across the tree of life for cellular functions including motion and sensation.

**Distal appendage**: projection at the distal centriole involved in membrane docking and ciliogenesis.

**Mature centriole**: a centriole of age greater than one cell cycle, which has disengaged from its parent, marked by cNap1 accumulation in mammals.

**Microtubule**: tubulin polymers nucleated by various centrosomal structures.

**Pericentriolar material**: protein coat of centrioles involved in microtubule nucleation that expands in size during mitosis.

**Procentriole:** a developing centriole early in its growth, generally attached perpendicularly to a more mature centriole.

**Proximal centriole**: the opposite end of a centriole to the distal appendages, sometimes containing a cartwheel structure from which procentrioles generally form.

**Rootlet**: fibrous and often striated cytoskeletal filaments found at centrioles. Here, I define rootlets as rootletin- or SF-assemblin-based striated centriolar fibres, but precise usage of the term varies dependent on field.

**Transition zone**: a zone at the base of cilia involved in entry to, and exit from, cilia.

Rootlets are well described by electron microscopy in eukaryotes from many different phyla (Table 1). There are fundamental similarities and differences in the architecture of centriole-associated structures found in different cell types across the tree of life (Yubuki and Leander, 2013). Here, I use the term rootlets to refer to all rootletin- or SF-assemblin-based striated fibres at centrioles (as discussed further below), while appreciating that structural, proteomic and functional differences exist in different organisms. Naming conventions differ between phyla; rootlets have variously been termed kinetodesmal fibres (Allen, 1969), the centrosome linker (Bahe et al., 2005; Mayor et al., 2000; Yang et al., 2006), interconnecting fibres, fibrous roots (Andersen et al., 1991; Horridge and Gray, 1965) and striated fibres (Kalnins and Porter, 1969; Lechtreck and Melkonian, 1998).

**Table 1.**
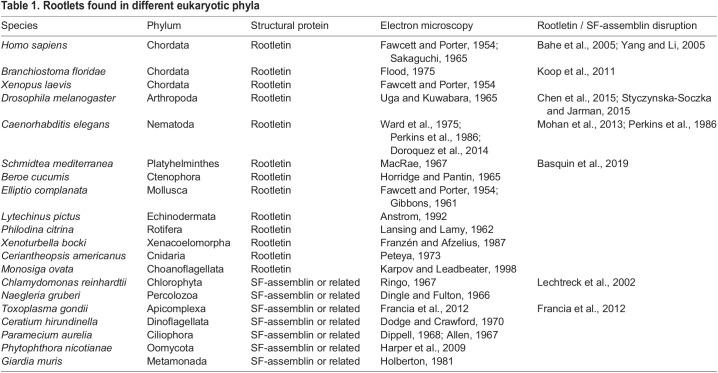
Rootlets found in different eukaryotic phyla

Rootlet size and morphology are variable – a theme found throughout different species. For example, in humans, rootlets are found in both ciliated and non-ciliated cells, where their length is ∼1–2 µm or up to tens of microns respectively in different cell types (Fig. 1B,C) (Anderson, 1972; Fawcett and Porter, 1954; Gilliam et al., 2012; Uzbekov et al., 2012). They may consist of a single fibrous structure, have a branched morphology, or be entirely absent (Anderson, 1972; Fawcett and Porter, 1954; Hagiwara et al., 1997; Stephens, 1975).

A major evolutionary conserved constituent of rootlets in the Animalia kingdom is rootletin protein (also known as ciliary rootlet coiled-coil protein, which in humans is encoded by the *CROCC* gene) (Yang et al., 2002). Rootletin forms macromolecular structures across the Animalia, according to sequence similarity, localisation studies and mutagenesis screens (Table 1). Rootletin is essential for rootlet formation in human cells (Bahe et al., 2005), flies (Chen et al., 2015; Styczynska-Soczka and Jarman, 2015), worms (Mohan et al., 2013), lancelets (Koop et al., 2011) and mice (Yang et al., 2002, 2005). Outside of the Animalia kingdom, rootlets in unicellular protists, such as *Tetrahymena thermophlia*, *Paramecium tetraurelia, Toxoplasma gondii* or *Chlamydomonas reinhardtii*, are formed from different proteins to rootletin, notably the SF-assemblins and related proteins (Lechtreck and Melkonian, 1991; Nabi et al., 2019; Soh et al., 2020). In this work, I focus discussion on rootletin-based and SF-assemblin-based rootlets.

There has been no systematic characterisation of all rootlet component proteins – for example by proteomic or microscopic methods. However, in addition to rootletin and SF-assemblins, ∼100 other proteins have been implicated in rootlet biology across different species, for differing reasons including localisation to rootlets by imaging and the phenotypic consequences of their disruption. These additional protein components are not discussed in detail here. Throughout this Review, I discuss functional similarities between rootlets in diverse life forms, while recognising that similarities could be the result of either common ancestry or convergent evolution.

## Positioning and linking multiciliary arrays

Rootlets are found widely and advances in genetics have allowed their targeted disruption; so what precisely are their functions?

### Rootlets maintain centriolar positioning and ciliary beating in multiciliated arrays

**Fig. 2.**
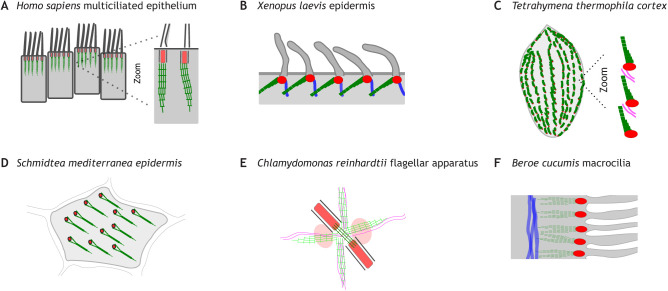
**Rootlets position and link multiciliary arrays.** (A) Cartoon representation of rootlets within multiciliated human epithelium, here the oviduct epithelium. Based in part on data in Hagiwara et al. (1997). Rootlets are shown in green. (B) *Xenopus* multiciliated epithelial cell in the skin, containing rootlets oriented opposite to the ciliary beat direction and linking to actin networks (shown in blue). (C) Unicellular protists including *Tetrahymena thermophila* and *Paramecium tetraurelia* have multiciliated arrays containing basal bodies separated by rootlets, which reach between nearest neighbours in the cortex. The cartoon illustrates a whole cell, with the oral apparatus depicted on the left-hand side. The cell cortex shows several layers of organisation which are not depicted here for simplicity. Rootlets are also called kinetodesmal fibres in this context. (D) Planaria such as *Schmidtea mediterranea* have chevron-shaped polarised rootlets in ventral epidermal multiciliated cells. (E) *Chlamydomonas reinhardtii* rootlets emanate from the flagellar apparatus. Two types of rootlet are shown in different shades of green, between the mature basal bodies and next to microtubules (shown in grey). These two types of *Chlamydomonas reinhardtii* rootlets are commonly referred to as the distal connecting striated fibre and the striated microtubule-associated fibres, respectively. (F) Macrocilia of the Beroidae family of ctenophores are used for feeding, and they contain rootlets extending from centrioles into actin filaments. Based on data in Tamm and Tamm (1987) from *Beroe cucumis*. The cartoons are simplifications of the morphology of the systems shown, see references in the main text for detailed information. Rootlets are shown in green and centrioles in red, actin in blue and microtubules in magenta.

Multiciliated cells generate fluid flow in numerous biological contexts, such as the swimming of single-celled organisms or mucociliary clearance in human airway epithelia (reviewed in Brooks and Wallingford, 2014). Multiple cilia can show coordinated behaviours to create directional motion, such as metachronal beating (see Glossary) (Sanderson and Sleigh, 1981). This requires mechanisms to orient and coordinate beating cilia. Rootlets are conspicuous by their presence in multiciliated cell types. In humans, they are prominent in multiciliated epithelial tissues, for example, in cells of the oviduct epithelium (Hagiwara et al., 1997) or brain ependyma (Klinkerfuss, 1964; Mahuzier et al., 2018), where they generally reach into the cell body from basal bodies (Fig. 2A).

Knockouts in various model systems have indicated that rootlets in multiciliated cells can contribute to the synchrony of ciliary movement and the positioning of basal bodies as part of polarised tissues. For example, *Xenopus laevis* embryo epidermis is covered with multiciliated cells in which rootlets form basal body arrays by linking each centriole to a sub-cortical actin network (Fig. 2B) (Antoniades et al., 2014; Park et al., 2008; Werner et al., 2011; Yasunaga et al., 2015). Changes to this network – by disruption of subapical actin – alters ciliary beating, such that instead of creating coordinated metachronal waves, cilia beat with a normal frequency but in a disorganised fashion (Werner et al., 2011). Unicellular ciliates including *Tetrahymena thermophlia* and *Paramecium tetraurelia* are covered by cilia that beat periodically. Arrays of rootlets are similarly present between nearest-neighbour basal bodies, associating closely with neighbouring centrioles and submembranous cytoskeletal structures at the cell cortex (Fig. 2C) (Allen, 1967, 1969; Iftode and Fleury-Aubusson, 2003; Nabi et al., 2019; Soh et al., 2020). Mutation of the *Tetrahymena* rootlet component *DisAp* disrupts ciliary beating and centrosomal positioning (Frankel and Jenkins, 1979; Galati et al., 2014; Jerka-Dziadosz et al., 1995). A third example comes from planarian flatworms, which have cilia on their ventral surface that beat in a synchronous manner for locomotion. Rootlets in planarians, such as *Schmidtea mediterranea*, are present between adjacent basal bodies in arrays aligned with the head-tail axis of the animal (Dorey, 1965; Rieger, 1981; Vu et al., 2019) (Fig. 2D). Knockdown of the rootlet-associated component VFL3 (also known as CCDC61) has no effect on motile cilia beat frequency, but disrupts the synchrony of ciliary movement and basal body positioning (Basquin et al., 2019).

There are many differences in the architecture and assembly of these diverse multiciliated systems. These observations together demonstrate a conserved theme, in which changes to both basal body positioning and coordinated ciliary beating occur after genetic disruption of rootlets.

### Resisting physical force beneath cilia

Rootlets have long been suggested to provide structural support to motile cilia, based primarily on their appearance extending into the cell body (Anstrom, 1992; Fawcett and Porter, 1954; Gibbons, 1961; Hard and Rieder, 1983; Holley, 1991). For example the *Beroidae* family of marine comb jellies (Ctenophores) use large ciliary organelles called macrocilia to rip apart prey (Horridge and Gray, 1965), and in this setting, rootlets extend from basal bodies into the cell to reach actin filaments (Fig. 2F) (Tamm and Tamm, 1987). The function of rootlets in macrocilia is not understood, but conceivably relates to the ability to resist or generate physical force.

How might rootlets provide anchorage to cilia in different biological settings? Force is possibly transmitted inside the cell from beating cilia to the basal body and associated structures (Bayless et al., 2016; Hard and Rieder, 1983). Metachronally beating flagella exert waves of pressure that induce undulations of the cell surface in some cases (Tamm, 1999). Rootlets are often found to be planar polarised in a direction that is opposite to that of the power stroke (see Glossary) of the cilium in multiciliated systems (Allen, 1969; Boisvieux-Ulrich et al., 1985; Gibbons, 1961; Mitchell et al., 2007). Rootlet disruption in *Tetrahymena* leads to basal body rotation in a manner that correlates with ciliary beating, consistent with cilia being capable of moving centrioles rotationally in the absence of anchorage in ciliates (Galati et al., 2014; Wright et al., 1983). One possibility is therefore that rootlets oppose the forces generated by motile cilia.

Physical force is a key pattern-forming parameter influencing cellular architecture within multiciliated tissue, in combination with factors including planar cell polarity signalling, the cytoskeleton and other centriole appendages (Wallingford, 2010). Cilia orientation is dynamically responsive to the direction of fluid flow in some cases (Marshall and Kintner, 2008; Mitchell et al., 2007), suggesting that force and planar polarity likely influence each other as part of a self-organising system containing feedback. Other centriolar appendages termed basal feet are also important. Basal feet orient in the opposite direction to rootlets (Sandoz et al., 1988), and similarly influence cilia orientation in multiciliated cells, in part through anchoring basal bodies to cytoskeletal networks (Anstrom, 1992; Basquin et al., 2019; Franzén and Afzelius, 1987; Gibbons, 1961, 1961; Hard and Rieder, 1983; Kunimoto et al., 2012; Sandoz et al., 1988; Steinman, 1968). A recent study suggests that a balance of force model, in which different forces – exerted by the actin and microtubule cytoskeletons – are balanced by the concerted anchoring of several centriole appendages (Basquin et al., 2019). Interestingly, both rootlets and basal feet appear capable of changing their structure in response to force in certain biological contexts (Liu et al., 2020; Soh et al., 2020). Ciliate rootlets change length and orientation depending on cilia-generated force, apparently to maintain centrosomal connections and cortical interactions over a timescale of hours (Galati et al., 2014; Soh et al., 2020). These observations suggest that centriolar appendages not only function to resist mechanical force, but also dynamically respond to it and convey it, to influence behaviours such as ciliary beating (Soh et al., 2020; Wan, 2018; Wolfrum, 1991). Overall, rootlets contribute to centriole positioning in multiciliated epithelia as part of integrated systems involving mechanical force, the cytoskeleton and other centriolar appendages.

Coordinated beating of multiple cilia depends on coupling of forces between adjacent cilia. Thus, adjacent cilia influence each other both hydrodynamically through movement of fluid in the extracellular space and intracellularly (Narematsu et al., 2015; Tamm, 1984; Wan, 2018). An untested theory is that vertebrate rootlets function as physical levers that facilitate propagation of the metachronal wave intracellularly, due to the connections they form between centrioles (Werner et al., 2011). Such a theory is reminiscent of a model from the green algae *Chlamydomonas reinhardtii,* which swims using two flagella, coupled by various different basal body-associated fibres (Hoops et al., 1984; Hyams and Borisy, 1975; Lechtreck and Melkonian, 1991; Ringo, 1967; Wright et al., 1983) (Fig. 2E). Mutation of the rootlet-associated component *vfl3* in this setting results in a loss of coordination of the ciliary beat strokes between neighbouring cilia, as well as basal body positional defects (Hoops et al., 1984; Wan and Goldstein, 2016; Wright et al., 1983). One theory is that the two flagella in *Chlamydomonas* are coupled oscillators, linked by rootlets (specifically the distal connecting fibres) (Guo et al., 2021; Klindt et al., 2017; Quaranta et al., 2015). According to this model, distal connecting fibres maintain ciliary beat synchronization in a fashion akin to the synchronization of Huygens' clocks – through the formation of physical links via which energy is transferred between different cilia (Guo et al., 2021; Klindt et al., 2017). There are many differences between the centriole-associated fibres of *Chlamydomonas* and vertebrate systems, such as the number of different types of rootlet, and the suggestion that some *Chlamydomonas* rootlets may be contractile (Geimer and Melkonian, 2004; Ringo, 1967; Wingfield and Lechtreck, 2018; Wright et al., 1983). It is therefore unclear whether similar principles could apply to multiciliated systems outside of biflagellated *Chlamydomonas*.

## Rootlets in sensory cilia

### Rootlets in mechanosensitive structures

**Fig. 3.**
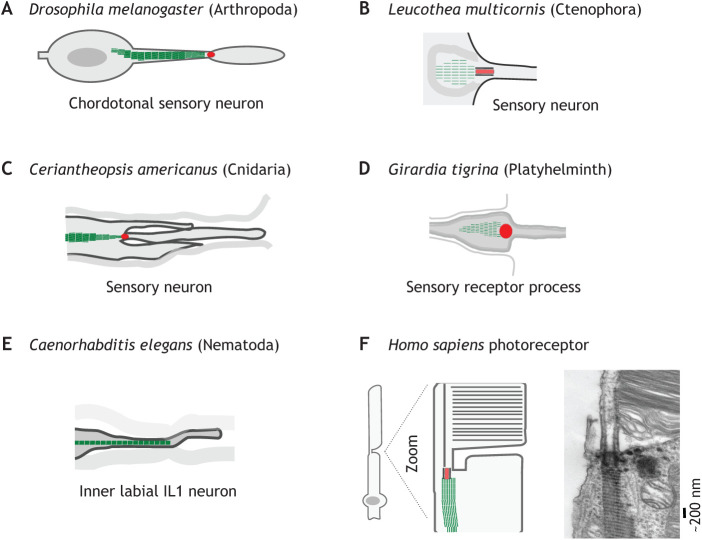
**Rootlets in sensory structures.** (A) *Drosophila* embryonic sensory neuron, part of a mechanosensing chordotonal organ (see Glossary). The rootlet is shown within a dendrite. (B) Onion-shaped rootlet found in the sensory neurons of a ctenophore, *Leucothea multicornis*. (C) Rootlet morphology in putative proprioceptors in the cnidarian *Ceriantheopsis americanus.* (D) Sensory receptor process of the platyhelminth *Girardia tigrina*. (E) Rootlet morphology in a *C. elegans* sensory neuron, part of the inner labial sensilla. (F) Electron micrograph and cartoon of rootlets in a *Homo sapiens* photoreceptor. Electron microscopy image provided by Dr Holger Jastrow, University of Duisberg-Essen (www.drjastrow.de), with permission. Rootlets are shown in green throughout.

Primary cilia are conserved components of mechanosensation underlying senses including touch, sound and proprioception (see Glossary), as well as developmental and homeostatic processes such as bone development (Malone et al., 2007; Xiao and Quarles, 2010). *Drosophila* rootlets are rod-like structures up to tens of microns in length, found in sensory neurons of the chordotonal and external sensory neurons (Keil, 1997; Wolfrum, 1992) (Fig. 3A). Rootletin knockout or knockdown in *Drosophila* causes impaired sensory neuron function, with multiple behavioural defects relating to mechanosensation (touch sensitivity, geotaxis and hearing) and chemosensation (gustatory perception) (Chen et al., 2015; Styczynska-Soczka and Jarman, 2015). Thus, rootlets are essential not just for the function of motile cilia, but also for the function of non-motile cilia involved in various sensory modalities in flies.

Rootlets are widely found in specialised mechanosensory cell types containing non-motile cilia in various species, where they can be large relative to the size of the cell. For example, marine invertebrate ctenophores use both the motile and sensory functions of cilia for much of their behaviour (Tamm, 2014). Sensory neurons on the surface of the ctenophore *Leucothea multicornis* – used to sense vibrations in surrounding water – have unusually shaped onion-like rootlets (Horridge and Pantin, 1965; Tamm and Tamm, 1991) (Fig. 3B). The cnidarian *Ceriantheopsis americanus,* in contrast, has elongated rootlets in putative proprioceptors – mechanosensory neurons mediating the sense of body movement (Peteya, 1973) (Fig. 3C). The planaria flatworm *Girardia tigrina* is another illustration of how rootlets can be found not just at motile cilia, since it contains distinct rootlets in both motile and non-motile cilia in sensory neurons (Fig. 3D) (MacRae, 1967).

Precisely why rootlets are required for the function of mechanosensory neurons has not been clearly elucidated in any species. The structure of sensory cilia appears largely normal following genetic removal of rootlets in flies (aside from the absence of rootlets themselves) (Chen et al., 2015; Styczynska-Soczka and Jarman, 2015), suggesting that they are not simply required for overall ciliary structure. One untested possibility is that since rootlets are large relative to the neuronal dendrites in *Drosophila* sensory neurons, they could be important for the gross mechanical integrity of mechanosensory cilia (Chen et al., 2015; Styczynska-Soczka and Jarman, 2015). A related untested possibility is that rootlets could be mechanically coupled to other ciliary structures to convey force to mechanosensitive ion channels. Mechanosensory neurons rely on mechano-gated ion channels to transduce mechanical stimuli into ionic currents (reviewed in Hehlert et al., 2021; Lumpkin and Caterina, 2007). NOMPC is one *Drosophila* mechanosensory ion channel; it is tethered to the microtubule cytoskeleton with ankyrin repeats, which act as elastic springs to mechanically gate the channel (Jin et al., 2017; Liang et al., 2013; Walker et al., 2000; Zhang et al., 2015). Since NOMPC is gated in this way, it raises the untested question of whether rootlets are involved in conveying force to these or other mechanosensitive channels, through forming intracellular links within the cell.

### Transport in specialised cilia

Like *Drosophila*, *Caenorhabditis elegans* has particularly large rootlets, up to tens of microns in length, in three ciliated sensory neurons (termed the IL1, OLQ or BAG inner labial mechanosensory neurons) (Doroquez et al., 2014; Perkins et al., 1986; Ward et al., 1975) (Fig. 3E). Two of these (IL1 and OLQ) are mechanosensory (Goodman, 2006; Hart et al., 1999; Perkins et al., 1986). Mutation of the *C. elegans* ortholog of rootletin (*CHE-10*) causes chemo-sensation defects, like in flies (Mohan et al., 2013; Perkins et al., 1986). However, in contrast to what is seen in flies, rootlet-knockout worms have a defect in the organization and function of the periciliary membrane compartment – an area of plasma membrane at the base of the cilium (Mohan et al., 2013). *CHE-10* mutant worms have age-dependent deterioration of sensory neurons and defects in intraflagellar transport (see Glossary) (Mohan et al., 2013; Perkins et al., 1986). Complicating these phenotypes, CHE-10 might, however, have additional functions that are separable from its rootlet functions (Chen et al., 2015; Mohan et al., 2013). First, *CHE-10* knockout leads to degeneration of neurons with or without notable rootlets (Mohan et al., 2013). Secondly, in contrast to rootletin in other systems, CHE-10 localises not just to ciliary rootlets, but also to the transition zone of cilia without rootlets (Chen et al., 2015; Mohan et al., 2013; Styczynska-Soczka and Jarman, 2015).

A frequent suggestion in the literature is that rootlets might provide a potential intracellular route for molecular motor-driven cargo (Fariss et al., 1997; Gilliam et al., 2012; Lechtreck et al., 2002; Mohan et al., 2013; Yang and Li, 2005; Yang et al., 2002). Indeed, in human photoreceptor cells, rootlets extend over 10 µm in length through the inner segment, hypothetically providing a direct route through the cell, and increasing the surface area of a centriole comparatively to a centriole without a rootlet (Fig. 3F) (Gilliam et al., 2012; Yang et al., 2002). Human photoreceptors are polarised neurons with a sensory cilium highly specialised for light detection (Liu et al., 2007), thus providing another example of large rootlets in sensation-associated primary cilia. The photoreceptor cilium is crucial for retinal homeostasis and development; defects in >50 centrosomal genes lead to inherited retinal dystrophies (Bujakowska et al., 2017). Genetically engineered mouse models with disruption of rootletin or its paralog cNap1 (also known as CEP250) show phenotypes including visual disfunction with reduced retinal thickness (Huang et al., 2019) and degenerative retinal function, respectively (Yang et al., 2002). In humans, mutations in cNap1 have been implicated in the degenerative eye disease retinitis pigmentosa (de Castro-Miró et al., 2016; Huang et al., 2019; Kumar et al., 2004), the hereditary progressive loss of rod photoreceptors and retinal pigment epithelial function in the eye. cNap1 mutations are also causative of atypical Usher syndrome, a ciliopathy (see Glossary), with visual impairment and hearing loss (Fuster-García et al., 2018; Khateb et al., 2014; Kubota et al., 2018).

Thus, rootlets appear to be important for photoreceptor function in mammals (Yang et al., 2005). Similar to the case in sensory neurons in flies and worms, the reasons for these associations between rootlet mutation and visual disfunction are unclear. It has been suggested that rootlets could contribute to high levels of protein traffic into the photoreceptor outer segment (Engelmann, 1880; Fariss et al., 1997; Gilliam et al., 2012; Yang and Li, 2005). Vesicles of unknown identity, and the molecular motor kinesin-1, have been found associated with rootlets in photoreceptors (Fariss et al., 1997; Gilliam et al., 2012; Yang and Li, 2005; Yang et al., 2002). However, there is no evidence of directed traffic along rootlets (Yang and Li, 2005) or mechanistic insight into whether photoreceptor rootlets directly participate in transport into the outer segment. An alternative explanation is that photoreceptor rootlets anchor the outer segment (Yang et al., 2005), akin to their putative structural roles in other settings (as discussed above). In support of this model, photoreceptors in rootletin gene targeted mice are vulnerable to experimentally applied mechanical stress (Yang et al., 2005). Together, these considerations highlight that rootlets are important in specialised sensory neurons, and yet their precise functions remain to be elucidated.

## Rootlet functions in non-ciliated cells

I have so far considered how rootlets function in motile and primary cilia. In invertebrates, such as *Drosophila* and *C. elegans*, rootlets do not decorate centrioles in non-ciliated cells, suggesting that their functions may be limited to ciliated cells in these organisms (Chen et al., 2015; Mohan et al., 2013; Styczynska-Soczka and Jarman, 2015). However, in other organisms, such as mammals, centrosomes not associated with cilia do also nucleate rootlets from both mature centrioles (Bahe et al., 2005; Bornens et al., 1987; Mahen, 2018; Paintrand et al., 1992).

Do rootlets have functions in non-ciliated cells? In mammalian cells, removal of rootlet components including rootletin results in the loss of centrosome cohesion, which is the spatial proximity of mature centrioles (Bahe et al., 2005; Floriot et al., 2015; Mayor et al., 2000; Turn et al., 2021). Rootlets are therefore frequently called the centrosome linker in this role, and regarded as forming a proteinaceous link between centrioles (Bahe et al., 2005). Loss of centrosome cohesion has been associated with defects in cell migration and mitosis (Decarreau et al., 2017; Floriot et al., 2015; Panic et al., 2015). During mammalian mitosis, centrosomes separate precisely to form spindle poles, preceded by the splitting of centrioles, a process termed centrosome disjunction. Centrosome disjunction (see Glossary) coincides with disassembly of rootlets, in part through phosphorylation of target proteins by the kinase Nek2A (also known as NEK2) (Bahe et al., 2005; Fang et al., 2014; Faragher and Fry, 2003; Fry et al., 1998a,b; Hardy et al., 2014). Experimental changes to this process influence spindle orientation in ensuing mitoses (Decarreau et al., 2017; Mardin et al., 2013). The reader is directed to other detailed discussions on the molecular mechanisms of centrosome disjunction in mammalian interphase cells (Agircan et al., 2014).

**Fig. 4.**
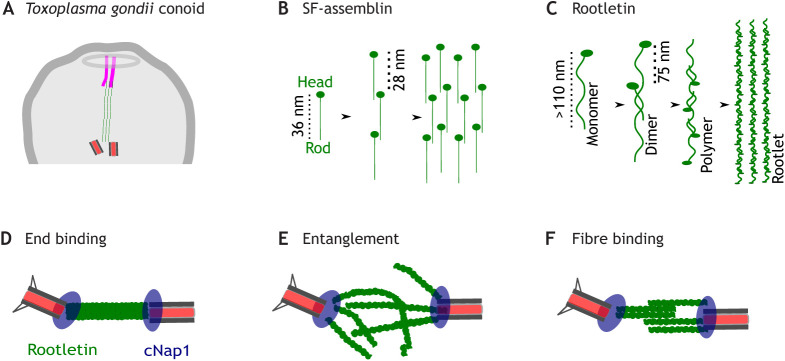
**Linking centrioles to other cellular structures.** (A) Cartoon of *Toxoplasma gondii* rootlet during cell division, connecting centrioles to a microtubule pair at the conoid. Based on Francia et al. (2012). SF-assemblin-based rootlets are shown in green and conoid microtubules in magenta. (B) A hypothetical SF-assemblin rootlet assembly model in protists, based on data in Lechtreck (1998) and Patel et al. (1992). SF-assemblin protofilaments form with a 28 nm periodicity from parallel dimers. The head domain is represented by a circle and the rod domain by a line. (C) A hypothetical rootlet assembly model in mammalian cells. Rootletin forms parallel dimers, which then assemble head-to-tail to form polymers. Polymers assemble further into rootlets. The scheme shown here is based in part on data from Ko et al. (2020) and Vlijm et al. (2018). (D) End-binding model of centrosome cohesion in mammalian cells. Cohesion is mediated by binding of rootletin fibre termini to cNap1 localised at both proximal centrioles in trans. (E) Entangling model. Cohesion is mediated by the entanglement of rootlets from opposing centrioles, restraining the movement of individual polymers. (F) Fibre binding model. Cohesion is mediated by specific interactions between rootletin fibres from opposing centrioles.

Another example of rootlet function outside of ciliated cells comes from apicomplexan parasites, which have SF-assemblin-based rootlets but generally do not have flagella (Lechtreck, 2003). Rootlets in the Apicomplexan *Toxoplasma gondii* physically connect centrioles to a structure involved in cell invasion called the conoid at the tip of the forming daughter cell during cell division (Fig. 4A) (Francia et al., 2012)*.* Remarkably, rootlets are spatiotemporal organisers of cell division in this context, because they directly form links that are required for genome and organelle segregation (Francia et al., 2012).

Together, these considerations highlight rootlet functions in non-ciliated cells, through the formation of physical links between centrioles and other cellular structures.

## Linking centrioles to other cellular structures

### Rootlet polymerisation

What are the molecular mechanisms by which rootlets form physical links within the cell? Since biopolymers frequently have hierarchical architectures that create properties underlying their functions, understanding how rootlet fibre subunits spatially arrange is one facet of this question.

As noted above, SF-assemblin is the major known constituent of one type of rootlet (often called the striated microtubule-associated fibres) in green algae including *Chlamydomonas* and *Spermatozopsis similis* (Lechtreck and Melkonian, 1991). SF-assemblin-based rootlets are thought to be mechanically rigid and biochemically stable, consistent with a function in maintaining stability of the basal apparatus (Lechtreck and Melkonian, 1991). SF-assemblin has a non-helical head domain and an α-helical rod domain that has the capability to form coiled-coils (Weber et al., 1993). The rod domain has consecutive 29-residue motifs, which consist of four heptads followed by a skip residue (Lechtreck, 1998). Protofilaments form *in vivo* and *in vitro,* with a diameter of 2 nm, an overall polarity and the capacity to form layers (Lechtreck and Melkonian, 1991; Patel et al., 1992). Striations are present in SF-assemblin rootlets at 28 nm intervals, possibly due to overlapping of 36 nm-long proteins (Fig. 4B) (Lechtreck, 1998; Weber et al., 1993).

Mammalian rootlets have long half-lives, are resistant to high concentrations of salt and are diffusionally stable over many hours during interphase (Bahe et al., 2005; Fry et al., 1998a; Mahen, 2018; Mardin et al., 2010; Yang et al., 2002). A combination of data, including from overexpression studies, and electron microscopy, super resolution and second-harmonic imaging microscopy, indicates that rootletin and Cep68 polymerise to form rootlet fibres (Akiyama et al., 2017; Bahe et al., 2005; Vlijm et al., 2018; Yang et al., 2002). Electron-dense striations are generally observed at 50–70 nm intervals in rootletin-based rootlets (Anderson, 1972; Fawcett and Porter, 1954; Gilliam et al., 2012; Uzbekov et al., 2012). Rootletin is a long fibrous protein, localising in rootlets with a repeating organization every 75 nm as shown by super resolution imaging or electron microscopy (Hagiwara et al., 1997; Sahabandu et al., 2019; Vlijm et al., 2018). Rootletin structural data is limited, but recent crystallographic information on the human rootletin R3 region (residues 1108–1317) shows that it forms a left handed parallel coiled-coil homodimer (Ko et al., 2020), stabilised by hydrophobic interactions and covalent bonds. One model is that rootletin α-helical chains intertwine their coiled-coil domains to form elongated dimers (Yang et al., 2002), which then further assemble into higher order multimers (Fig. 4C). Vlijm et al. (2018) suggest a head-to-tail staggered rootletin polymer, interspersed with Cep68. Cep68 binds to rootletin filaments every 75 nm via its C-terminus, which contains a conserved spectrin repeat (Man et al., 2015; Vlijm et al., 2018). Another untested possibility is that adjacent rootletin polymers bind through their coiled coils (Ko et al., 2020). However, most details of rootlet polymerisation are unknown, including the direction of association of sequences, the site of subunit addition, potential assembly intermediates and the identity of the electron-dense striations.

### Forming organelle–organelle contacts

Rootlets have been found closely associated with various structures by microscopy, such as Golgi stacks (Coulon et al., 1986; Mazo et al., 2016; Tenkova and Chaldakov, 1988), mitochondria (Olsson, 1962), the nuclear envelope (Potter et al., 2017), the cell cortex (Allen, 1969; Iftode et al., 1996), intermediate filaments (Sandoz et al., 1988) and actin filaments (Hard and Rieder, 1983; Tamm and Tamm, 1987). A common theme in multiciliated systems is the association of rootlets with microtubules or the actin cytoskeleton. For example, in *Chlamydomonas*, one type of rootlet (the striated microtubule-associated fibres) is spatially close to microtubules (reviewed by Lechtreck and Melkonian, 1998). Similarly, in *Tetrahymena*, rootlets associate with microtubule appendages on neighbouring centrioles, via electron-dense linkages of unknown identity (Allen, 1967; Soh et al., 2020). In general, however, details of putative molecular complexes allowing such associations are unclear. A possible exception is in *Xenopus*, where rootlets are suggested to be linked to subapical actin through focal adhesion complexes that include paxillin and focal adhesion kinase (Antoniades et al., 2014). This is an intriguing suggestion given that focal adhesions have the capability to sense force (del Rio et al., 2009).

Furthermore, interactions between rootletin and the nesprin 1α isoform have been found in photoreceptors and ependymal cells, perhaps suggesting that this interaction docks rootlets to the nuclear envelope (Potter et al., 2017). This observation of a nucleus–cytoplasmic link is reminiscent of nucleus–basal body connections formed by rootlets in protist flagellates, such as *Chlamydomonas* and the amoeboflagellate *Naegleria gruberei.* Often termed rhizoplasts (or system II fibres) in this context, one rootlet end adheres to basal bodies and the other terminus ends in an invagination of the nuclear envelope (Dingle and Fulton, 1966; Salisbury et al., 1984). It should be noted that protists in general have more rootlet types than human cells, however, with nucleus–basal body connectors formed from centrin rather than SF-assemblin (Salisbury et al., 1984).

### Models of centrosome cohesion in mammalian cells

In mammalian cells, siRNA knockdown and gene knockout has shown that cNap1 is required for attachment of rootlets to proximal centriole ends (Bahe et al., 2005; Yang et al., 2006), suggesting that it might anchor rootlet fibres to centrioles (see Box 1 for definition of proximal centriole). Centriole disengagement is the separation of an immature centriole from its parent at the end of mitosis. Mammalian rootlets are not present on procentrioles, and cNap1 loading onto the newly disengaged proximal centriole is hypothesized to allow rootlet formation in interphase (Fry et al., 1998a; Hardy et al., 2014; Mahen, 2018; Mayor et al., 2000; Tsou and Stearns, 2006). How the proximal centriole is organized to nucleate rootlets is unclear. This is an important open question, since it relates both to the spatiotemporal control of rootlet formation and more generally to the principles underlying centrosome assembly. One possibility is stereospecific interactions, consisting of cNap1-binding sites spatially arranged into a ring shape (Vlijm et al., 2018) onto which rootletin or Cep68 attach. This would make cNap1 functionally equivalent to the γ-tubulin complexes that form microtubules (Moritz et al., 2000), perhaps arranging rootletin into a nucleation-competent orientation or concentration. Electron microscopy suggests that rootlets attach centrally to the centriole in ciliated osteocytes (Uzbekov et al., 2012). Cep135 is found in the centriolar lumen, is required for centrosome cohesion and binds to cNap1, so it is possible that cNap1 attaches to it there (Hardy et al., 2014; Kim et al., 2008; Sonnen et al., 2012; Tian et al., 2021). However, it is notable that, based on electron microscopy observations, rootlets appear to attach to multiple different locations on centrioles, either in the centriole lumen or at the side of the barrel (Hagiwara et al., 2008).

Although rootletin has been suggested to form links between centrioles in mammalian cells, the precise molecular nature of these links are unclear. One untested possibility is that cNap1 – which is found at both centrioles – binds to each rootlet terminus in trans (Fig. 4D) (Yang et al., 2006). This model entails end-on binding of rootlets to centriole barrels, an orientation which is visible in electron microscopy of insect scolopodia rootlets (Jana et al., 2018; Keil, 1997). In centrosome preparations from cultured cells, centriole pairs have been found to be linked by filaments at their proximal ends (Bornens et al., 1987; Paintrand et al., 1992). However, live-cell imaging has revealed that centrosome cohesion is dynamic, with centrioles at times transiently separating (Au et al., 2017; Bahe et al., 2005; Piel et al., 2000), and it is possible that only a limited subset of centriole orientations are sampled in fixed-cell techniques. In agreement with this possibility, rootlets have been shown to occupy various orientations relative to the centriole–centriole axis, one of which is radial rootlet orientation distally from centrioles (Bahe et al., 2005; Lauweryns and Boussauw, 1972; Mahen, 2018; Sahabandu et al., 2019; Vlijm et al., 2018). Electron microscopy of primary cilia in rat osteocytes (Uzbekov et al., 2012) and in the oviduct (Hagiwara et al., 2008) shows that rootlets from the central region of one centriole contact the outer wall of the other centriolar cylinder. Accordingly, a different model of centrosome cohesion posits that rootlets from each centriole non-specifically entangle (Bahe et al., 2005) (Fig. 4E). The details of this model are unknown. Polymers at high density constrain each other according to reptation theory (see Glossary; de Gennes, 1971), whereby chain diffusion coefficient decreases linearly with the length of the polymer. It is, however, unclear whether rootlets are long or dense enough to maintain centrosome cohesion by reptating. Since rootletin polymers apparently associate with each other to form rootlets, another alternative is that rootlets from different centrioles could also maintain centrosome cohesion via dynamic lateral or end-on binding of the fibres (Ko et al., 2020; Yang et al., 2002) (Fig. 4F). In support of this model, fibres from neighbouring centrioles have been observed by electron microscopy to laterally associate with their cross striations in phase (Fawcett and Porter, 1954; Vlijm et al., 2018), and two different filaments have been seen to merge together to remain associated in live-cell imaging experiments (Mahen, 2018). It is clear that in various cell types rootlets originating from different centrioles associate closely with each other, suggesting that this could be a general mode of interaction. Overall, these considerations hint at diverse orientations among rootlets, perhaps indicating that the mechanisms of centrosome cohesion are both dynamic and cell type specific. Further future investigation of centrosome cohesion might provide a basis to understand how rootlets form links between different cellular structures, perhaps with dynamic and variable interactions.

## Perspective – rootlets and the organisation of cellular architecture

I have considered rootlet functions across diverse species and contexts. A universal theme is the capability of rootlets to mediate physical contacts between centrioles and other cellular structures. In some instances, these contacts relate to the formation of subcellular pattern formation – such as the maintenance of centriolar spacing and positioning in multiciliary arrays. In other cases, rootlet functions are intimately associated with the dynamic cellular response to physical stress, whether it originates from beating cilia, the interphase cytoskeleton or in specialised mechanosensory neurons. It is possible that physical requirements within cells have incentivised rootlet evolution as structures able to resist or convey force, in parallel to other cytoskeletal systems. In this regard, rheology to understand the response of rootlets to force could be informative in the future. Future work could also probe the putative molecular interactions that mediate linkages between rootlets and other cellular structures, as well as systematically characterize which other cellular structures rootlets are capable of directly contacting. Related to this, understanding of how rootlet components assemble into filaments is hampered by a lack of structural data. Rootletin or SF-assemblin crystal structures, *in vitro* studies on subunit packing within fibres or cryo-electron tomography of purified rootlets could be informative in the future. This could provide insight into emergent rootlet properties that are important for function, such as size and nanomechanics. Together, addressing these issues will aid the understanding of both rootlet structure and function, and how collective organelle function results in the generation of cellular processes.
